# Questioning central assumptions of the ICAP framework

**DOI:** 10.1038/s41539-023-00197-4

**Published:** 2023-11-15

**Authors:** Christian M. Thurn, Peter A. Edelsbrunner, Michal Berkowitz, Anne Deiglmayr, Lennart Schalk

**Affiliations:** 1 ETH Zurich, Zurich, Switzerland; 2 LMU Munich, Munich, Germany; 3 Universität Leipzig, Leipzig, Germany; 4 Pädagogische Hochschule Schwyz, Schwyz, Switzerland

**Keywords:** Education, Education, Psychology

## Abstract

Closing the research-practice gap in education is an important aim. The ICAP framework (for interactive, constructive, active, and passive engagement modes) explicitly targets this aim and has gained broad attention. The ICAP framework is supposed to support practitioners in translating research findings into practice by distinguishing between four modes of student engagement. In this comment, we consider two central assumptions of the ICAP framework. First, the four modes of engagement are assumed to be “reflected in the overt behavior the student exhibits while undertaking an activity”^1^, and thus observable for teachers. Second, the ICAP framework assumes that the interactive mode of engagement is most effective for learning, followed by constructive, then active, and lastly passive modes (i.e. I > C > A > P, the so-called ‘ICAP-hypothesis’^1,2^). We argue that both assumptions are inconsistent with central tenets of empirical educational research. First, it is not sufficient to rely on overt behaviors as indicators of learning, because they are ambiguous with respect to the underlying learning process and do not reliably indicate them. Second, there is no “one size fits all”-order of engagement modes. Supposedly inferior engagement modes excel when used in the right way, on the right learners, and with the right timing regarding the learning process. We elucidate the use of formative assessment to gain insight into covert learning processes. Whereas the ICAP framework provides a seemingly plausible and easily actionable guide for practice, practitioners should not be advised to rely on ICAP for selecting effective interventions and assessing learning processes in the classroom.

## Questioning central assumptions of the ICAP framework

Translating insights from educational research into tangible and applicable guidelines for practitioners (e.g. teachers) is of major importance. Researchers have tried to overcome the research-practice gap by developing freameworks that are both accessible and provide clear guidelines (e.g. ref. ^3^). A widely known framework is the ICAP framework proposed by Chi and colleagues^1,2,4^. The ICAP framework postulates four modes of engagement, from which its acronym was derived: the *Interactive*, *Constructive*, *Active*, and *Passive* mode. These four modes of engagement are thought of as hierarchical, with higher modes comprising lower ones, and are differentiated on the basis of students’ overt behaviors. For example, simply reading a text would be indicative of the *passive* mode, underlining text passages while reading would be indicative of the *active* mode, generating self-explanations on the text would be indicative of the *constructive* mode, and discussing a text with a learning partner would be indicative of the *interactive* mode. The reason for the hierarchical ordering is that the four observable modes of engagement are assumed to be a good-enough heuristic for non-observable, that is, covert learning processes. “While recognizing that such mapping cannot be perfectly accurate 100% of the time, it might be good enough for an authentic classroom environment”^2^. Thus, the ICAP framework links students’ overt behaviors to covert cognitive processes and learning outcomes, and it proposes that “higher” modes of engagement (I > C > A > P) come with a higher probability of sustainable learning^1,2,4^. According to Chi and Wylie^1^ “the ICAP framework has strong practical implications as teachers and other instructional designers can use it to choose, modify, or design tasks for students to perform”.

The goal of this comment is to critically discuss two core assumptions of the ICAP framework. The first is that different modes of cognitive engagement are to “be detected by overt behaviors”^2^. The second is that the four engagement modes are hierarchically ordered regarding their effectiveness for learning, with the interactive mode being the most effective^1,2^. We agree with the authors of the ICAP framework that communicating to practitioners on how to support students’ learning is of utmost importance. However, we argue that these two assumptions of the ICAP framework—detection via overt behaviors and hierarchical ordering of engagement modes—likely lead to wrong inferences in practice, and do not correspond well with research on effective learning and instruction. We first outline these two points of criticism, shortly scrutinizing their empirical basis. Note that this comment is not aimed at systematically reviewing empirical research on the ICAP framework. Rather, we take a look at specific studies that have been reported as strong support for the ICAP framework, and we bring up general insights from the last decades of educational research that question the validity of the framework. Afterward, we discuss potential steps forward.

## Is overt behavior a reliable indicator of students’ cognitive engagement?

The four modes of cognitive engagement of the ICAP framework focus on the visible features in student behavior (i.e. overt behavior in instructional events, such as reading a text, taking notes, or discussing). Chi and colleagues do acknowledge that the “correspondence between overt behavior and cognitive processes is not perfect”^4^. For example, students might covertly process content deeply while appearing passive. Their claim is that the link between overt behavior and cognitive processes is probabilistic rather than deterministic, but that most of the time the correspondence holds well enough^2,4^. Is this really the case?

Educational research has repeatedly demonstrated that the covert features, rather than the overt features of student behavior, determine the effectiveness of learning activities (e.g. refs. ^3,5,6^). Such covert features encompass, for example, attending and understanding the lesson, problem-solving, or relating new information to prior knowledge^7^. Models of instructional quality, summarizing factors that research has shown to be conducive to learning, acknowledge this distinction and abstain from focusing on overt features of students’ activities (e.g. ref. ^3^). Rather, established evidence-based models emphasize factors such as effective classroom management, the activation of meaningful cognitive processes in learners, and their individual support^8^. Although Chi and colleagues^4^ purportedly agree with this argument, the ICAP framework and its predecessor DOLA (Differentiated Overt Learning Activities^1^) categorize students’ learning processes based primarily on overt student behavior. Chi^9^ argues that attending to overt behaviors is a good approach, as overt behaviors often are the only information that practitioners can observe, overt behaviors can be sufficiently differentiated, and overt behaviors can help align activities and goals. As noted, Chi acknowledges the limitations of this assumption in stating that the correspondence between overt behavior and covert learning processes is only probabilistic. They add that “if necessary, students’ […] engagement modes can be discriminated more precisely by comparing the information contained in students’ outputs with the information contained in the instructional materials”^2^. Yet, it is worrying that research applying the ICAP framework ignores these caveats and equates overt behavior with cognitive engagement in a simplified manner, relying generally on overt behavior to identify the learning process (e.g. refs. ^10–14^). That is, putting the ICAP framework into practice seems to entail the danger of relying on the misunderstanding that overt behavior alone is sufficient for gaining insight into students’ learning processes.

Let us provide some examples demonstrating that equating overt behavior with cognitive engagement is fallible. First, Chi (e.g. refs. ^2,4^) argues that watching a video would fall in the passive mode category. However, watching a video while following an observation assignment (e.g. paying attention to a specific event in the video that is crucial for understanding a key concept) can bring students into a mode that is cognitively active (by deeply engaging with the key event) or even constructive (by relating the key event to prior knowledge), even though the overt behavior remains identical. Second, a student copying examples from the board would be seen as being in an active mode of engagement (e.g. refs. ^2,4^). Whereas the copying task very likely induces procedural engagement, it is not visible from watching the students whether they are cognitively engaged so that the task will be conducive to learning. In comparison, a student listening to a teacher explaining the relevance of examples on the blackboard, without copying them, would appear passive, but may indeed learn better (see e.g. ref. ^15^ on relevance instruction; and^16^ on expansive framing). Further research has shown that tasks involving copy-and-paste-like writing procedures are not very conducive to learning^17^, whereas appropriate teacher guidance is a critical element in any kind of learning activity^18^. Put more generally, empirical evidence from different perspectives suggests that it is the covert effects that influence how students learn. The heuristic to equate overt engagement with learning is not only misleading, it might even distract teachers from implementing instructional features that are actually conducive to learning. This critique is supported by recent research that reflects on the term “active learning”^19^. In line with our view, these researchers argued that overt behavior can be misleading regarding the identification of covert cognitive processes. Moreover, if “engagement with instructional materials can be operationalized as active if some form of overt motoric action or physical manipulation is undertaken”^1^, this operationalization may add a barrier to including students with disabilities^19^. Therefore, relying primarily on overt behavior to plan, monitor, and judge the effectiveness of instructional events and students’ learning in the classroom, even if it is regarded only as a heuristic, bears the risk of distorting insights into the actual covert learning processes.

Other frameworks of learning have long acknowledged this problem and handle the term active learning carefully. For example, Reinmann-Rothmeier and Mandl^20^ regarded active learning strictly as the triggering of cognitive processes. Such triggering—sometimes referred to as cognitive activation (e.g. ref. ^21^)—may or may not be related to overt behavior. Consequently, changes in knowledge components (i.e. the covert learning processes) can only be detected via deliberate assessment (e.g. ref. ^3^). Put differently, assessment events are necessary to gain insight into students’ learning processes.

To sum up, overt behavior is an unreliable proxy of the cognitive activities of students and of the effectiveness of instruction (e.g. refs. ^5,6^). This evaluation is also congruent with research on engagement arguing that although behavioral and cognitive engagement can be related, their relation is so uncertain that they should not be mistaken for one another^22^.

Chi and colleagues^1,2,4,9^ acknowledge that the link between observed behaviors and cognitive engagement is weak and suggest attending to students’ products: “behavioral engagement, along with student products, jointly, may be an adequate (but not perfect) measure to reflect the differentiated underlying cognitive processes that students are undertaking”^4^. They suggested that teachers need to evaluate students’ outputs, “on the rare occasions when teachers need an accurate resolution of such ambiguities“^2^. Chi and colleagues define student output as all the products that are resulting from the learning task, “such as explanations from self-explaining, notes from note-taking, hypotheses from inducing, questions from question-asking, predictions from generating, concept maps from drawing, self-report assertions such as ‘I don’t understand’ from monitoring, perhaps in the context of other utterances such as problem-solving protocols^9^. However, it does not become clear when and in which situations teachers are supposed to take outputs into account. In addition, as described in the articles about the ICAP framework, student output is not a diagnostic, structured product. Producing output during a learning task mainly has the goal to be conducive to learning, but whether it is diagnostic of the learning process is unclear.

## Is the hierarchy of modes of engagement a useful heuristic?

To the best of our knowledge, there has been no systematic evaluation of the hierarchy of modes of engagement as the central hypothesis derived from the ICAP framework (i.e. I > C > A > P; with ‘>’ indicating more successful learning). Chi and Wylie^1^ explicitly state that their review of evidence is selective and that it only refers to research in favor of the framework. The studies put forward as the strongest tests of the ICAP hierarchy^2,23^ have several weaknesses and lack ecological validity. In the first study with 42 undergraduate engineering students, Menekse and colleagues^23^ used a within-group design with one engagement mode for the whole day alongside other activities of the course. We highlight four weaknesses of this study. First, an artificial focus on one engagement mode lacks validity for the varied forms of instruction in authentic classrooms over a complete school day (for a similar criticism see refs. ^24,25^). Real classroom instruction follows carefully designed sequences of different kinds of learning tasks that relate to and build upon one another, rather than isolated activities; for example, sequences of collaboration, problem-solving and direct instruction (see e.g. refs. ^26,27^). Such sequencing, which is ubiquitous in educational practice, also contradicts the assumption of one engagement mode that is always superior; rather, the effectiveness of a specific learning activity depends on the timing and the readiness of the students. Second, Menekse and colleagues^23^ measured learning of different content with different learning tasks after each day, preventing direct comparisons of the effectiveness of a mode by topic. However, there might be interactions between learning content and instructional strategies. Third, the interactive and constructive modes showed a negligible and non-significant difference, which does not support the ICAP hypothesis (see also ref. ^25^). Fourth, this study did not test the three conditions for their superiority over the passive mode. Thus, this first study does not provide strong support for the ICAP hypothesis.

In the second laboratory study, Menekse et al.^23^ investigated a sample of 120 undergraduate students using a between-group pre-posttest design, including all four engagement modes. In contrast to cognitive-educational research emphasizing the need to enrich learning activities with activating prompts^17^, learners in the passive condition had to read aloud text, producing an artificial task that undermines basic self-regulation strategies such as the rereading of difficult passages^28^. Overall, the methodology of both studies are far away from real classroom practice. They lack ecological validity that is necessary to close the research-practice gap and the results do not provide unequivocal support for the ICAP hypothesis.

Similar to the first study of Menekse et al.^23^, other studies have found that the hierarchy of the ICAP framework does not hold; for example, Conley and colleagues^12^ report higher levels of engagement for passive activities compared to active activities. Studies not explicitly relying on the ICAP framework also do not fit to the suggested hierarchy. For example, in a series of studies concerned with algebra learning in sixth graders, Ziegler et al.^29^ found that direct instruction (encompassing more overtly passive student engagement and less constructive student activities) resulted in better learning outcomes than carefully designed self-learning materials (encompassing more overtly active and constructive modes of engagement). These findings are in accordance with the well-established principle that particularly novice learners benefit from more strongly teacher-guided instruction^18^, which may be dismissed by an ICAP-trained observer as more passive and thus inferior instruction. To summarize, the empirical basis for the hierarchy specified in the ICAP framework is not convincing.

## A step forward: harnessing formative assessment to uncover covert learning processes

We agree with Chi and colleagues that practitioners need evidence-based tools that are practical in everyday teaching to get insight into students’ learning processes. Fortunately, there are reliable and valid approaches that serve this aim. One prime candidate is formative assessment: systematic and regular assessment activities before, during, and after instruction^30–32^. In contrast to the ICAP framework, formative assessment acknowledges that there is no simple way to monitor learning processes such as just noticing learners’ overt behaviors and sometimes looking at their products. Accepting that learning processes cannot be observed directly^5^, formative assessment encompasses repeated targeted assessments of learning outcomes that provide insight into covert learning processes. Information from these assessments provides teachers and learners with feedback and guidance to optimize teaching and learning processes. Formative assessment done regularly^30^ can yield deep insights into students’ covert learning processes, conceptions, and level of processing. In contrast to the inconclusive empirical basis which is available for the ICAP hierarchy, meta-analyses on formative feedback support its effectiveness (e.g. refs. ^31,32^).

To promote learning, practitioners need to think about students’ (cognitive) activities and learning processes, define learning goals, devise suitable assessment techniques, provide feedback, and check the alignment of these steps in combination with their explanations and guidance during instruction^33^. As an anonymous reviewer of this commentary wrote: “Teachers need to evaluate whether the research-informed practices they are implementing are having a positive impact on their learners in their context”. The ICAP framework might be misleading towards this aim as modes of engagement are judged by two susceptible sources of information: overt behaviors and student output (“products”) resulting from learning tasks (e.g. notes while solving problems). Learning tasks have the primary demand to be conducive to learning, to help learners practice, to develop their conceptual understanding, to challenge them, or sometimes to make them fail (e.g. ref. ^34^). Learning tasks are not explicitly designed to deliver diagnostic information. In formative assessment, the primary source of information is the assessment task. Other information (e.g. what students are doing) is used as a secondary source of information to get an overall diagnostic assessment. Formative assessment creates an explicit assessment situation and is (usually and mostly) framed accordingly. Decades of empirical evidence suggest that systematic formative assessment helps to gain reliable insight into the complex interplay of instruction and learning^30–32^.

In sum, we argue that observing whether a student is watching a video, taking notes, writing summaries, or discussing with other students is not sufficiently reliable to gain insight into learning processes. It is crucial to systematically monitor and assess the covert learning processes to reach a certain learning goal. The ICAP framework, however, does not provide guidance on how to generate and use such diagnostic information on students´covert learning processes. In contrast, the systematic use of formative assessment techniques provides a flexible, scalable, generalizable, and evidence-based toolbox for practitioners. Systematic use of formative assessment may put a higher demand on practitioners’ shoulders than following the ICAP framework, but it will provide them with more valuable and valid information that they can use to flexibly adapt instruction to the classroom and effectively support students’ learning.

### Reporting summary

Further information on research design is available in the Nature Research Reporting Summary linked to this article.

### Supplementary information

Reporting Summary

## References

[1] Chi MT, Wylie R (2014). The ICAP framework: linking cognitive engagement to active learning outcomes. Educ. Psychol..

[2] Chi MT (2021). Translating a theory of active learning: an Attempt to close the research‐practice gap in education. Top. Cogn. Sci..

[3] Koedinger KR, Corbett AT, Perfetti C (2012). The knowledge-learning-instruction (KLI) framework: toward bridging the science-practice chasm to enhance robust student learning. Cogn. Sci..

[4] Chi MT, Adams J, Bogusch EB, Bruchok C, Kang S (2018). Translating the ICAP theory of cognitive engagement into practice. Cogn. Sci..

[5] Klieme EEmpirische (2006). Unterrichtsforschung: aktuelle entwicklungen, theoretische grundlagen und fachspezifische befunde. Einführung in den thementeil. Z. Fur. Padagog..

[6] Seidel T, Prenzel M, Rimmele R, Dalehefte IM, Herweg C (2006). Blicke auf den physikunterricht. Ergebnisse der IPN videostudie. Z. Fur. Padagog..

[7] Peterson PL, Swing SR, Braverman MT, Buss RR (1982). Students’ aptitudes and their reports of cognitive processes during direct instruction. J. Educ. Psychol..

[8] Kunter M, Klusmann U, Baumert J, Richter D, Voss T (2013). Professional competence of teachers: effects on instructional quality and student development. J. Educ. Psychol..

[9] Chi MT (2009). Active‐constructive‐interactive: a conceptual framework for differentiating learning activities. Top. Cogn. Sci..

[10] Antonietti, C., Schmitz, M. L., Consoli, T., Cattaneo, A., Gonon, P. & Petko, D. Development and validation of the ICAP technology scale to measure how teachers integrate technology into learning activities. *Comput. Educ*. 10.1016/j.compedu.2022.104648 (2023).

[11] Barlow A, Brown S (2020). Correlations between modes of student cognitive engagement and instructional practices in undergraduate STEM courses. Int. J. STEM Educ..

[12] Conley Q, Sadauskas J, Christopherson R, Lin L, Ilgaz H (2022). Facebook usage patterns looking into the mind via the ICAP engagement framework. Behav. Inf. Technol..

[13] Goldberg P, Sümer Ö, Stürmer K, Wagner W, Göllner R (2021). Attentive or not? Toward a machine learning approach to assessing students’ visible engagement in classroom instruction. Educ. Psychol. Rev..

[14] Sailer M, Stadler M, Schultz-Pernice F, Franke U, Schöffmann C (2021). Technology-related teaching skills and attitudes: Validation of a scenario-based self-assessment instrument for teachers. Comput. Hum. Behav..

[15] McCrudden MT, Schraw G (2007). Relevance and goal-focusing in text processing. Educ. Psychol. Rev..

[16] Engle RA, Lam DP, Meyer XS, Nix SE (2012). How does expansive framing promote transfer? Several proposed explanations and a research agenda for investigating them. Educ. Psychol..

[17] Dunlosky J, Rawson KA, Marsh EJ, Nathan MJ, Willingham DT (2013). Improving students’ learning with effective learning techniques: promising directions from cognitive and educational psychology. Psychol. Sci. Public Interest.

[18] Kirschner P, Sweller J, Clark RE (2006). Why unguided learning does not work: an analysis of the failure of discovery learning, problem-based learning, experiential learning, and inquiry-based learning. Educ. Psychol..

[19] Lombardi D, Shipley TF (2021). & Astronomy team, biology team, chemistry team, et al. The curious construct of active learning. Psych. Sci. Public Interest.

[20] Reinmann-Rothmeier, G., & Mandl, H. Lehren im Erwachsenenalter. Auffassungen vom Lehren und Lernen. In *Psychologie der Erwachsenenbildung* 4th edn, Vol. 1 (*Eds*. Weinert, F. E., Mandl, H.), Ch. 355-403 (Hogrefe, Göttingen, 1997).

[21] Blömeke S, Jentsch A, Ross N, Kaiser G, König J (2022). Opening up the black box: teacher competence, instructional quality, and students’ learning progress. Learn. Instr..

[22] Sinatra GM, Heddy BC, Lombardi D (2015). The challenges of defining and measuring student engagement in science. Educ. Psychol..

[23] Menekse M, Stump GS, Krause S, Chi MT (2013). Differentiated overt learning activities for effective instruction in engineering classrooms. J. Eng. Educ..

[24] El-Mansy SY, Barbera J, Hartig AJ (2022). Investigating small-group cognitive engagement in general chemistry learning activities using qualitative content analysis and the ICAP framework. Chem. Educ. Res. Pract..

[25] Wiggins, B. L., Eddy, S. L., Grunspan, D. Z., & Crowe, A. J. The ICAP active learning framework predicts the learning gains observed in intensely active classroom experiences. *AERA Open*10.1177/2332858417708567 (2017).

[26] Sinha T, Kapur M (2021). When problem solving followed by instruction works: Evidence for productive failure. Rev. Educ. Res..

[27] de Jong, T., Lazonder, A. W., Chinn, C. A., Fischer, F., Gobert, J. et al. Let’s talk evidence–the case for combining inquiry-based and direct instruction. *Educ. Res. Rev*. 10.1016/j.edurev.2023.100536 (2023).

[28] Mayer RE, Moreno R (2003). Nine ways to reduce cognitive load in multimedia learning. Educ. Psychol..

[29] Ziegler E, Edelsbrunner PA, Stern E (2018). The relative merits of explicit and implicit learning of contrasted algebra principles. Educ. Psychol. Rev..

[30] Black P, Wiliam D (2009). Developing the theory of formative assessment. Educ. Assess. Eval. Account..

[31] Graham S, Hebert M, Harris KR (2015). Formative assessment and writing: a meta-analysis. Elem. Sch. J..

[32] Lee H, Chung HQ, Zhang Y, Abedi J, Warschauer M (2020). The effectiveness and features of formative assessment in US K-12 education: a systematic review. Appl. Meas. Educ..

[33] Biggs J (1996). Enhancing teaching through constructive alignment. High. Educ..

[34] Kapur M, Bielaczyc K (2012). Designing for productive failure. J. Learn. Sci..

